# Prenatal exposure to ambient air pollution and autism spectrum disorders: Results from a family‐based case‐control study

**DOI:** 10.1002/jcv2.12129

**Published:** 2022-12-16

**Authors:** Nima Ghahari, Fatemeh Yousefian, Ehsan Najafi

**Affiliations:** ^1^ Centre for Health Services Research Faculty of Medicine University of Queensland Brisbane Queensland Australia; ^2^ Department of Survey Engineering Faculty of Civil Engineering Shahid Rajaee Teacher Training University Tehran Iran; ^3^ Department of Environmental Health Engineering Faculty of Health Kashan University of Medical Sciences Kashan Iran

**Keywords:** air pollution, autism, case‐control, family‐based, traffic‐related

## Abstract

**Background:**

Autism prevalence has increased considerably, but its etiology is still poorly understood. While there have been suggestions regarding associations between air pollution exposure and neurodevelopmental disorders, several studies have looked at the effect of air pollution exposure on autism. However, the results are inconsistent. The possible role of unknown confounders is mainly blamed for this inconsistency.

**Methods:**

To minimize confounding effects, we evaluated the impact of air pollution exposure on autism using a family‐based case‐control study. Cases were individuals with a diagnosis of autism born between 2009 and 2012 in Isfahan city, Iran. The controls did not have a previous history of autism and were cousins of the case person. The controls were matched with the autistic cases in terms of residential location and age range. For each trimester of pregnancy, carbon monoxide (CO), nitrogen dioxide (NO_2_), ozone (O_3_), sulfur dioxide (SO_2_), and PM_10_ exposure were estimated using the inverse distance weighted method.

**Results:**

The analysis indicates a significant association between CO exposure and autism in the second trimester (OR = 1.59; *p* = 0.046, 95% CI: 1.01–2.51) and entire pregnancy (OR = 2.02; *p* = 0.049, 95% CI: 1.01–2.95). Likewise, exposure to NO_2_ during the second trimester (OR = 1.17; *p* = 0.006, 95% CI: 1.04–1.31), third trimester (OR = 1.11; *p* = 0.046, 95% CI: 1.01–1.24), and entire pregnancy (OR = 1.27; *p* = 0.007, 95% CI: 1.07–1.51) were found to be associated with increased risk of autism.

**Conclusions:**

Overall, our study found higher exposure to CO and NO_2_, particularly during the second and third trimesters of pregnancy, was significantly associated with a higher risk of autism.

**Key points**

- Recent studies provide evidence on the more critical role of environmental factors on autism.
- Some studies examined the association between air pollution exposure and autism; however, the results are inconsistent.
- The possible role of unknown confounders and differences in genetic background are mainly blamed for this inconsistency.
- We examined the impact of exposure to air pollution on autism in a family‐based case‐control study to minimize the role of confounders.
- Our results implicate a significant association between exposure to traffic related air pollution and autism, especially in pregnancy's second and third trimesters.

## INTRODUCTION

Autism is a serious neurodevelopmental disorder characterized by varying degrees of deficit in verbal and nonverbal communication and social interaction, mostly accompanied by repetitive behavioral patterns (American Psychiatric Association, 2013). As there is no known cure for autism, this results in lifelong impacts on the child and family (Ghahari et al., 2022; Van Heijst & Geurts, 2015).

The number of children diagnosed with autism has increased during recent decades (Chiarotti & Venerosi, 2020; Maenner et al., 2020). Improvements in autism diagnosis and recognition, as well as inclusion of milder cases, have contributed to higher incidence numbers; however, this alone does not fully explain this notable rising trend of autism diagnosis (Hertz‐Picciotto & Delwiche, 2009; Lyall et al., 2017).

Based on recent evidence from Autism and Developmental Disabilities Monitoring ADDM research in the USA, one in every 54 individuals suffers from autism (Maenner et al., 2020). The high rate of prevalence makes autism one of the most frequent neurodevelopmental disorders in the world (Fombonne, 2009).

Despite the high prevalence rate, the etiology of autism is poorly understood (Ghahari et al., 2021; Tick et al., 2016). Early studies suggest the high heritability of autism and as a result, researchers have focused on identifying fundamental genetic causes (Bailey et al., 1995; Betancur, 2011). However, novel innovations and large population‐based studies provide evidence about the possible role of environmental factors on autism (Hallmayer et al., 2011; Sandin et al., 2014). Examples of these can be seen in twin studies. Twin studies mainly compare dizygotic twins (share 50% of their genes) with monozygotic twins (share 100% of their genes) and provide the ability to distinguish the contribution of gene and environmental factors. The amount of heritability reported in early twin studies varies considerably and ranges between 64% and 91% (Tick et al., 2016). However, recent studies report more moderate genetic heritability and provide evidence that the environment's impact is up to 40%–50% (Deng, Zou et al., 2015; Hallmayer et al., 2011).

This evidence has motivated many researchers to examine the association between different environmental risk factors and autism. However, although air pollution exposure has been found to be associated with various adverse birth defects (Girguis et al., 2016; Stieb et al., 2012) and childhood neurological disorders (Becerra et al., 2013; Guxens et al., 2012), limited studies have examined the association between autism and exposure to air pollution (Chun et al., 2020). In addition, there is a contradiction between the results of conducted studies. For example, while findings from studies conducted in the USA, Taiwan, and Israel indicate significant associations between air pollution exposure and autism (Becerra et al., 2013; Jung et al., 2013; Kalkbrenner et al., 2015; Raz et al., 2015; Talbott et al., 2015; Volk et al., 2013), European studies found no association (Gong et al., 2014, 2017; Guxens et al., 2016). These differences mainly result from the impact of unknown confounders such as genetic diversity and different responses to environmental risk factors between different genes. These differences are simply ignored in traditional case‐control studies (Yousefian et al., 2018). Although family‐based studies would minimize the impact of unrealized confounders and bring more similarities in genetic background, to the best of our knowledge, no study has examined the impact of air pollution on autism using this approach. To address this gap, we evaluated the associations between autism and exposure to ambient air pollution during each trimester of pregnancy and early infancy in a family‐based case‐control study. Cases and controls in this study are the children of two sisters, one of whom has autism and the other has no signs of autism.

## METHODS

### Research location

Our investigation is conducted in Isfahan, the third‐largest city in Iran. Isfahan is a big, homogenized city with fewer immigrants and less genetic and socioeconomic diversity than the capital and other big cities of Iran (Shahnam et al., 2010). Isfahan is located in the center of Iranian Plateau within longitude of 52.36° E and latitude of 33.28° N. Isfahan has a semi‐arid climate with an annual mean temperature of 16°C and mean precipitation of 125 mm Being the most industrialized city in Iran, the air of Isfahan is vastly affected by industrial emissions and motor traffic, especially during stagnant conditions.

### Study design and participants

#### Cases

The study population included 97 children diagnosed as having autism with severe difficulties in verbal and nonverbal communication, repetitive behavioral patterns and narrow interests. These children were born between 2009 and 2012 to mothers residing in the 15 counties of Isfahan city during their pregnancy and their child's early infancy. We engage the cases in cooperation with the State Welfare Organization of Isfahan. Pediatricians predominantly did the first recognitions, but it is verified by census among psychiatrists in the state welfare organization of Isfahan. The census has based their diagnosis on the Diagnostic and Statistical Manual of Mental Disorders‐IV, Text Revision (DSM‐IV‐TR). This census has classified children based on their functioning and severity of symptoms. The children with low functioning were selected for receiving governmental financial support and interventional services. The cases we chose come from this group of children with severe symptoms of autism. After identifying the cases, questionnaires were completed through a phone interview with mothers of children. Twenty‐three cases were excluded because (a) their residential location has changed during pregnancy (18); or (b) refused to participate in the study (5). The remaining 74 cases entered our analyses.

#### Controls

Fundamental trouble in referral‐based studies is identifying controls from similar underlying population matched with cases. These difficulties are addressed in family‐based studies through which controls are matched genetically and demographically to cases. In this study, we have asked the mother enrolled to contact their sisters and ask if they would like to be contacted by the researchers for potential study inclusion. Those who met the required qualifications and agreed to participate were then contacted for questionnaire completion. The qualification for controls to enter the examination was that they (a) had to have no previous history of autism; (b) were cousins of the case person, (c) their mother inhabited in Isfahan during the whole pregnancy, (d) they were born between 2009 and 2012. We excluded 21 cases because their residential location had been changed or they were not residents of Isfahan during pregnancy. Overall, 54 controls were included in our analysis.

### Covariates

The following covariates, which have been found to be associated to autism in previous studies, were collected by questionnaires: (a) Child characteristics (i.e., date of birth, sex, birth order, type of birth, child's gestational age in weeks, and birth weight); (b) Parental characteristics (i.e., age at delivery, education, cousin marriage, parental smoking, and maternal disease during pregnancy); (c) Family history of autism; and (d) residential location during pregnancy.

### Exposure assessment

**FIGURE 1:**
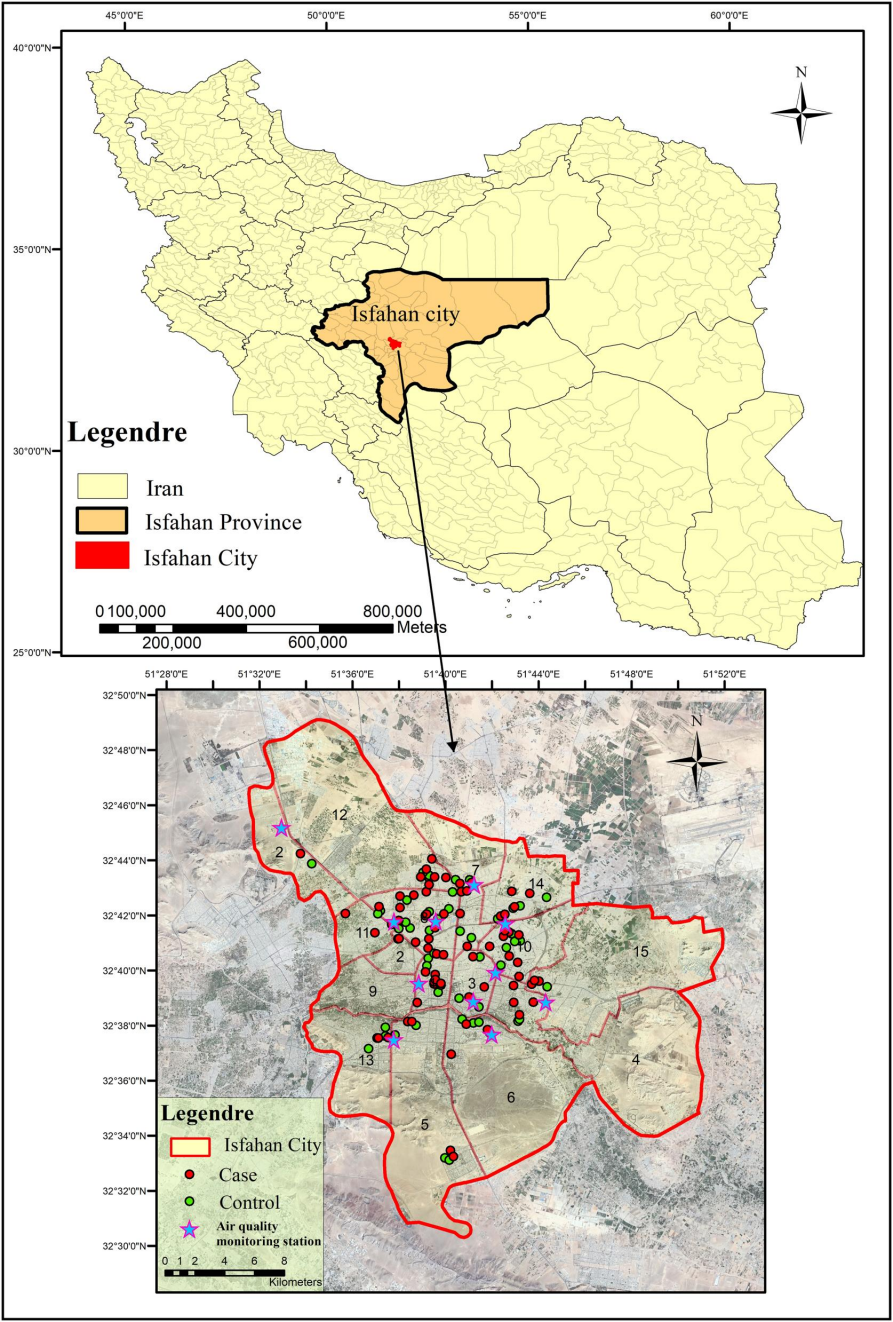
Spatial distribution of cases and controls.

The air quality data were obtained from 11 air‐monitoring stations belonging to Isfahan Municipality and Environmental Protection Organization (EPO) (Figure 1). This monitoring data included hourly measurements of carbon monoxide (CO), nitrogen dioxide (NO_2_), ozone (O_3_), sulfur dioxide (SO_2_), and PM_10_. The monitoring data were integrated into an average of 15 days point data and inverse distance weighted interpolation method (IDW) was used for providing air pollution surfaces from these point data (Deng et al., 2016; Deng, Lu et al., 2015). Using this method, 24 pollution surfaces for every year were built. Our IDW approach used spatial resolution (50 m) and the inverse distance‐squared weighted average of the three closest stations to compute the mean concentration. After building pollutant surfaces, each participant's location and time period of pregnancy was used for estimating their exposure to air pollution. This information was obtained through the data collection form. Two of the main parts of our data collection forms were questions regarding the participant's residential address during pregnancy and information regarding their child's birth date and gestational age. The addresses provided from the questionnaire were used to obtain latitude and longitude location coordinates. ArcGIS 10.2 was used for geocoding purposes. In order to accelerate the success rate of geocoding process, addresses were checked manually. Ninety‐four percent of the 128 eligible cases and controls were geocoded successfully, and the remaining were re‐standardized and re‐submitted for the geocoding process. After geocoding the participants' residential addresses, the time period of each trimester of pregnancy was calculated based on the child's gestational age and birth date. Using these air pollution surfaces, residential locations, and time periods, the individuals' average air pollution exposure for each trimester of pregnancy and early infancy estimated. Spatial distribution of air pollution stations, autism cases and their familial controls are provided in Figure 1.

### Statistical analysis

SPSS (version 23) was used for the statistical analysis. The univariate logistic regression model was used to examine the impact of variables and detect the potential confounders. The relation between air pollution exposure and autism were examined in two different logistic regression models: one crude logistic regression and one adjusted logistic regression with adjustment of effect of variables with *p*‐value <0.2 (type of birth, child's gestational age, parental age at delivery, paternal education, cousin marriage, and history of autism in family).

## RESULTS

Table 1 lists the characteristics of cases and controls. In our study, the majority of children diagnosed with autism were boys (83.8%). Likewise, boys account for 83.4% of matched controls. We found a significant association between autism and cousin marriage. While only 11.1% of controls were born from cousin parents, the rate of cousin marriage between cases was 40.5%. Moreover, there was a significant association between higher paternal age and autism prevalence (*p*‐value = 0.003). Caesarean section and gestational age were comparatively lower in cases (Table 1).

**TABLE 1:** Spatial distribution of cases and controls

Variable	Cases (%)	Controls (%)	*p*‐Value
All participants (128)	74	54	
Child characteristics			
Gender			
Girl	12 (16.2%)	9 (16.6%)	0.946
Boy	62 (83.8%)	45 (83.4%)	
Birth order			
First	47 (63.5%)	29 (53.7%)	0.588
Second	19 (25.7%)	20 (37%)	
Third	6 (8.1%)	4 (7.4%)	
Forth or more	2 (2.7%)	1 (1.9%)	
Birth weight in gram			
<2500	7 (9.5%)	4 (7.4%)	0.905
2500–3000	25 (33.8%)	14 (25.9%)	
3000–3500	26 (35.1%)	22 (40.7%)	
3500–4000	16 (21.6%)	10 (18.5%)	
>4000	0 (0%)	4 (7.4%)	
Type of birth			
Vaginal delivery	26 (35.1%)	12 (22.2%)	0.117
Caesarean section	48 (64.9%)	42 (77.8%)	
Childs' gestational age in week			
<37	13 (17.6%)	3 (5.6%)	0.054
≥37	61 (82.4%)	51 (94.4%)	
Parental characteristics			
Maternal age at delivery			
<20	6 (8.1%)	2 (3.7%)	0.133
20–25	20 (27%)	9 (16.7%)	
25–30	33 (44.6%)	23 (42.6%)	
30–35	8 (10.8%)	15 (27.8%)	
>35	7 (9.5%)	5 (9.3%)	
Paternal age at delivery			
<20	1 (1.4%)	3 (5.6%)	0.003
20–25	2 (2.7%)	14 (25.9%)	
25–30	24 (32.4%)	15 (247.8%)	
30–35	23 (31.1%)	16 (29.6%)	
>35	24 (32.4%)	6 (11.1%)	
Maternal education			0.553
<High school	44 (59.5%)	27 (50.0%)	
High school	7 (9.5%)	7 (13.0%)	
College degree	23 (31.1%)	20 (37.0%)	
Paternal education			0.117
<High school	52 (70.3%)	30 (55.6%)	
High school	9 (12.2%)	6 (11.1%)	
College degree	13 (17.6%)	18 (33.3%)	
Cousin marriage			
Yes	30 (40.5%)	6 (11.1%)	0.001
No	44 (59.5%)	48 (88.9%)	
Maternal smoking			
Yes	1 (1.4%)	1 (1.9%)	0.822
No	73 (98.6%)	53 (98.1%)	
Paternal smoking			
Yes	15 (20.3%)	8 (14.8%)	0.429
No	59 (79.7%)	46 (85.2%)	
Maternal disease			
Yes	8 (10.8%)	5 (9.3%)	0.774
No	66 (89.2%)	49 (90.7%)	
Family history of autism			
Yes	9 (12.2%)	1 (1.9%)	0.063
No	65 (87.8%)	53 (98.1%)	

**FIGURE 2:**
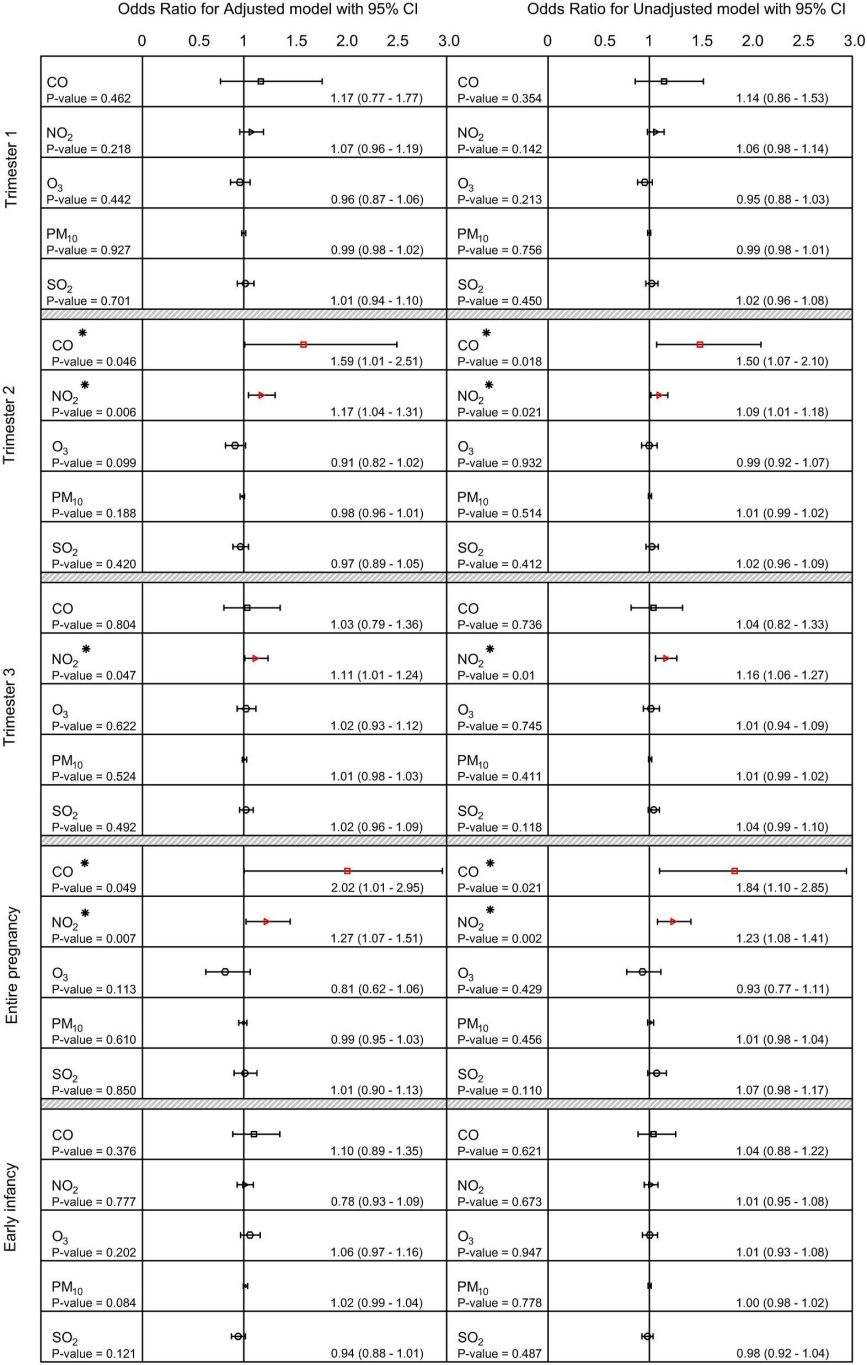
Odds ratios of association between autism and exposure to air pollutants.

The amount of individuals' exposure to CO, NO_2_, O_3_, PM_10_, and SO_2_ was estimated using IDW model for every trimester of pregnancy and early infancy. The average exposure to these ambient air pollutions is available in Table 2. Furthermore, the results of examining association between air pollution exposure and autism in adjusted and unadjusted logistic regression are provided in Figure 2. In unadjusted logistic regression model exposure to CO was significantly associated with autism in second trimester (OR = 1.50; *p* = 0.018, 95% CI: 1.07–2.0) and entire pregnancy (OR = 1.84; *p* = 0.021, 95% CI: 1.10–2.85). As well as this, autism prevalence was significantly associated with exposure to NO_2_ in second trimester (OR = 1.09; *p* = 0.021, 95% CI: 1.01–1.018), third trimester (OR = 1.16; *p* = 0.010, 95% CI: 1.06–1.27), and entire pregnancy (OR = 1.23; *p* = <0.001, 95% CI: 1.08–1.41) (Table 2).

**TABLE 2:** On average exposure to ambient air pollutants between cases and controls

		Mean (SD)				Mean (SD)	
		Cases	Controls			Cases	Controls
CO (ppm)	Trimester 1	6.1 (1.4)	5.9 (1.0)	NO_2_ (ppb)	Trimester 1	37.7 (4.8)	36.4 (4.6)
	Trimester 2	6.4 (1.6)	5.8 (1.0)		Trimester 2	38.4 (5.9)	36.2 (4.0)
	Trimester 3	6.1 (1.4)	6.0 (1.5)		Trimester 3	38.4 (5.3)	35.3 (4.0)
	Entire pregnancy	6.2 (0.7)	5.9 (0.7)		Entire pregnancy	38.1 (3.9)	35.9 (2.8)
	Early infancy	6.7 (1.8)	6.5 (1.9)		Early infancy	37.9 (5.3)	37.5 (5.4)
O_3_ (ppb)	Trimester 1	33.8 (4.4)	34.8 (5.0)	PM_10_ (ppm)	Trimester 1	155.6 (23.0)	156.9 (22.9)
	Trimester 2	33.1 (4.5)	33.2 (4.6)		Trimester 2	154.5 (24.4)	151.7 (22.1)
	Trimester 3	34.7 (4.6)	34.4 (5.0)		Trimester 3	158.5 (24.8)	155.2 (20.2)
	Entire pregnancy	33.9 (1.8)	34.1 (2.2)		Entire pregnancy	156.2 (12.2)	154.6 (11.8)
	Early infancy	33.8 (4.4)	33.8 (5.2)		Early infancy	160.5 (22.2)	159.4 (24.3)
SO_2_ (ppb)	Trimester 1	31.3 (5.5)	30.5 (6.6)				
	Trimester 2	30.5 (5.8)	29.6 (6.0)				
	Trimester 3	31.8 (6.5)	29.9 (7.1)				
	Entire pregnancy	31.2 (3.8)	30.0 (4.5)				
	Early infancy	30.6 (6.1)	31.4 (6.4)				

To ensure that covariates did not affect our evaluation, an adjusted logistic regression model was implemented. The associations after adjusting for variables with *p*‐value <0.2 did not change significantly and the results of the adjusted model was similar to the results of the crude model. Similar to the results of prior analysis, there was a significant association between CO exposure and autism in second trimester (OR = 1.59; *p* = 0.046, 95% CI: 1.01–2.51) and entire pregnancy (OR = 2.02; *p* = 0.049, 95% CI: 1.01–2.95). Moreover, the association between NO_2_ exposure and autism in second trimester (OR = 1.17; *p* = 0.006, 95% CI: 1.04–1.31), third trimester (OR = 1.11; *p* = 0.046, 95% CI: 1.01–1.24), and entire pregnancy (OR = 1.27; *p* = 0.007, 95% CI: 1.07–1.51) remained significant (Figure 2).

## DISCUSSION

The findings of our study from both adjusted and unadjusted models estimate 10 ppb increase in NO_2_ exposure during the second trimester, third trimester, and entire pregnancy would cause 9.4%–17%, 11%–16%, and 23%–17% relative increase in risk of autism respectively. Moreover, 50%–59% and 84%–102% relative increase in risk of autism for 1 ppm increase in CO exposure during the second trimester and entire pregnancy in both adjusted and unadjusted models were observed (Figure 2).

The association between air pollution and autism has been observed in several studies. The study of Volk et al. (2011) was the first study that suggests exposure to traffic‐related air pollution during pregnancy may increase the risk of autism (Volk et al., 2011). This study compared children living in close proximity of freeways to those living further away and found that autism was strongly associated with living <309 m of the freeways (OR = 1.86, 95% CI: 1.04, 3.45). Similarly, studies from the United States, Israel, and Taiwan provide evidence on the positive association between air pollution and autism (Becerra et al., 2013; Jung et al., 2013; Kalkbrenner et al., 2015; Raz et al., 2015; Talbott et al., 2015; Volk et al., 2013), which is inconsistent with the result of European studies (Gong et al., 2014, 2017; Guxens et al., 2016). The lack of association between autism and air pollution in European studies seems to have resulted from several factors. First, the comparatively lower amount of air pollution reported in European studies. For example, while European studies estimated the mean level of PM_10_ and NO_2_ exposure to be 10 μg/m^3^ and 17–44 μg/m^3^ respectively (Burman & Norman, 2013; Guxens et al., 2016), other studies either from the USA or Asia report comparatively higher exposure at approximately 25–36 μg/m^3^ for PM_10_ and 32–58 μg/m^3^ for NO_2_ (Becerra et al., 2013; Jung et al., 2013; Volk et al., 2013). Likewise, estimated air pollution exposure in our study was higher than in European studies (Table 2). Second, differences in the case definition. While American and Asian studies based their case eligibility on the diagnosis of autism, two‐thirds of European studies selected children with autism traits as cases (Guxens et al., 2016). The Diagnosis and Statistical Manual of Mental Disorders, fourth edition, text revision was based on the diagnosis of autism in our study, and our cases mainly included children with severe signs of autism. Third, differences in residual confounding from socioeconomic factors may have influenced the result of the analysis. For example, a Swedish study found an association between lower socioeconomic status and higher risk of autism (Rai et al., 2012), opposite to the USA findings (Thomas et al., 2012). As we have recruited relative controls in this study and cases are well‐matched to controls on genic and other risk factors, the significant association between air pollution and autism is less likely to be confounded by unknown family‐based variables. Moreover, unlike previous studies, the result of our analysis after adjusting for confounders did not change (Becerra et al., 2013; Kalkbrenner et al., 2015; Raz et al., 2015; Talbott et al., 2015). This stability provides evidence of more regulated matching between cases and controls.

This study found exposure to CO and NO_2_, particularly during the second and third trimesters of pregnancy, is associated with an increased risk of autism. This is in line with the findings of several other studies (Jung et al., 2013; Volk et al., 2013). CO and NO_2_ are two of the main pollutant surrogates of traffic exposure, and exposure to these pollutants can reasonably and logically be attributed to proximity to traffic (Liu et al., 2019; World Health Organization, 2021). Association between proximity to traffic and autism prevalence has been observed in several other studies as well (Gong et al., 2017; Volk et al., 2011, 2013). Clinical evidence indicates that prenatal exposure to some specific chemicals and toxins can interfere with the in utero process of brain development by stimulating early activation of the immune system and neuroinflammation (Neniskyte & Gross, 2017; Takano, 2015). This early activation of the immune system has been found to be associated with autism in both human and animal models of autism (Careaga et al., 2013; Gadad et al., 2013). Some studies found exposure to CO and NO_2_ may associate with immune dysregulation and neuroinflammation (Kalkbrenner et al., 2014; Kelly, 2003). However, others believe that these air pollutants are more likely to be surrogates of traffic‐related air pollution rather than a direct cause of neuroinflammation and early neuroimmune system activation (Kim et al., 2014; Wang et al., 2021). More clinical studies on examining the impact of more complex traffic‐related pollutants like secondary pollutants formed in the atmosphere and non‐combustion emissions on early activation of the immune system and neuroinflammation are strongly recommended.

### Strengths and limitations

Autism is a complex neurodevelopmental disorder resulting from the contribution of gene and environmental risk factors. Even though cases and controls come from the same residential location in traditional case‐control studies, these types of studies are highly likely to be manipulated by unknown variables. An example of these unknown confounders can be seen in differences in the genetic background between cases and controls and varying sensitivities to environmental pollution between different genes. In contrast to traditional case‐control studies, family‐based case‐control studies can mitigate the impact of genetic diversity and possible unrecognized confounders. As well as these, the family‐based study would lead to less bias in selecting controls. Relative controls are routinely more interested in participating, which would lead to more accurate answers and more reliable findings and results.

Despite these strengths, there are some noteworthy limitations. The most important of these limitations is the sample size restriction, which comes from the fact that there is no registration for autism in Iran. Our study included low‐functioning autistic children receiving government services and support. Another limitation that is common in many epidemiological studies with air pollution is considering outdoor air pollution estimation as the individual‐level exposure. Evidence indicates differences between indoor and outdoor air quality (Challoner & Gill, 2014; Chen & Zhao, 2011), and factors like indoor activities and ventilation conditions are the leading causes of these differences (Liu et al., 2006; Raunemaa et al., 1989). Although these factors may cause differences between indoor and outdoor air quality, several studies provide evidence of the interaction between indoor and outdoor air quality (Leung, 2015; Lonc & Plewa, 2011). These studies indicate that indoor air quality is highly affected by outdoor air pollution; likewise, indoor air pollutants affect outdoor air quality as well. Another limitation in terms of exposure assessment is the fact that some air pollution monitoring station in Isfahan has some days without data due to service interruptions. These limitations may adversely affect the accuracy and generalizability of our results.

## CONCLUSION

We evaluated the impact of prenatal exposure to air pollution on autism. A family‐based case‐control study was employed to minimize the role of unknown confounders. Overall, we found exposure to CO and NO_2_ associated with autism, especially during pregnancy's second and third trimesters. As exposure to these pollutants can be attributed to proximity to traffic, studies examining the association between more complex traffic‐related air pollution and autism prevalence are recommended. The findings of these studies would provide helpful information on understanding the etiology of autism and provide further prevention and remedial strategies.

## AUTHOR CONTRIBUTIONS

Nima Ghahari and Fatemeh Yousefian conceptualized and designed the study. Nima Ghahari performed data collection. Nima Ghahari and Fatemeh Yousefian performed analysis. Nima Ghahari wrote the first draft of the manuscript. Ehsan Najafi contributed to improving the manuscript and considering the editors' suggestions. All authors read and approved the final manuscript.

## CONFLICT OF INTEREST

The authors have declared that they have no competing or potential conflicts of interests.

## ETHICAL CONSIDERATIONS

All procedures performed in this study involving human participants were conducted in accordance with the Helsinki declaration and the institutional and national research committee's ethical standards. We contacted the cases and controls in collaboration and under the supervision of the state welfare organization of Iran. The study protocol, research plan and questionnaires were reviewed and approved by the organization's Research Ethics Committee prior to contacting the participants. All participants were informed of the study's purpose, the voluntary nature of participation, and their right to withdraw at any time.

## Data Availability

Individual participant data is not publicly available due to ethical restrictions. The unanimous version of the data is available upon reasonable request. Access to this data requires the approval of the State Welfare Organization of Iran.
